# Supplementary material for: The eye, a spy hole on human mind: Spontaneous blink rate and amplitude, and their variability, as new psychobiological markers of anxiety
Source: PLoS One. 2025 Dec 10;20(12):e0338262. doi: 10.1371/journal.pone.0338262 (PMC12694841; doi:10.1371/journal.pone.0338262)
Supplement: Attachment — Submitted filename: Rebuttal letter.docx [file pone.0338262.s003.docx]

**REBUTTAL LETTER**

PONE-D-25-09086

The eye, a spy hole on human mind: spontaneous blink rate and amplitude, and their variability, as new psychobiological markers of anxiety

**Reviewer # 1 Q1 -** While I don’t doubt the correlations the authors have found, I do question the value of the findings and the validity of the applications they envision. Below, I note that the main limitation of this manuscript is a failure to fully establish the theory of why the spontaneous blink would correlate to measures of anxiety. In addition, I do have concerns about the methodology, which was done post hoc. Because the authors did not originally intend to measure the spontaneous eyeblink, they did follow the typical protocols for doing so. Without replication, the correlation of one blink characteristic to state anxiety feels tenuous.

**Authors -** even if we have revised the ms. to improve its readability, we do not agree with the reviewer for several reasons: 1) we have explained the rationale of our study, starting from blink(startle) reflex-anxiety relationship and the literature, both in the intro and in the discussion. Now in the revision we have further explained 2) why we expected blink rate and amplitude to be correlated with state anxiety explaining how the neural hub modulated by anxiety for both startle and spontaneous blink is the reticular system (ARAS), 3) we have cited the previous paper showing a BR-anxiety relationship suggested the reviewer (it was published in the same month during which we submitted our paper, so we could not know it), 4) we had established the direction of the relationship and now this is more explicitly written, so that it should be clear that our study was hypothesis-driven (see also other points) and not explorative.

Concerning the methodology: ours were not post hoc analyses, we had completely new hypotheses and we carried out new analyses on a sample of data collected for different hypotheses and aims. This is exactly what data science is doing by re-analyzing past data from public repositories, that is, by reformulating new hypotheses on data collected in the past and reusable for different aims and hypotheses.

Concerning "without replication..the correlation....feels tenuous", most of the scientific literature does not involve replicated results but original results: without the publication of the original study is not possible to replicate it. Statistics serve to demonstrate that results are not random. We further clarified this issue at pg. 7 see below:

"For the purpose of the present research aimed at studying basic properties of blink activity, any convenience sample of healthy students used in past research was acceptable, as the original hypothesis for which the sample was recruited and the conditions to which it was submitted do not affect our aims and new hypotheses. This especially holds for resting state measures but also measures collected under emotional stimulation are in line with the need to distinguish correlation of blink rate with anxiety during resting state vs during emotional stimulation condition."

In addition, since the reviewer claimed that our results were "fairly weak" several times and we do not understand where there is this evidence as we discussed only significant statistics, we decided to report also the well known statistical concept that Pearson's correlation, besides significance, is also an index of the Effect Size and our effect sizes can be considered medium to large for most correlations (see below, and pg.12 highlighted in yellow):

"From another view, we can also consider the effect sizes of the links among our variables: indeed Person's correlation, in addition to the statistical significance which depends on sample size, is also an effect size, with r= 0.1-0.2 representing a small effect, r = 0.3 a medium effect, and r = 0.5 a large effect [26]. Thus, most of our effects ranged from medium (r= 0.288) to medium-large (r= 0.352) to large (r= 0.5 or higher).This provides further insight on the strength of our effects."

**Reviewer # 1 Q2 -** The authors review past literature (their own) showing that the amplitude of a reflexive, startle blink correlates with state but not trait anxiety measures. Given the nature of their paradigm, which used an acoustic startle stimulus, this relationship makes sense. However, the authors then argue that the overlap between the circuitry of the spontaneous blink and the startle blink predicts that the spontaneous blink should show the same relationship with measures of anxiety. In addition, given the relative ease of measuring the spontaneous blink, they note that it might be ideal compared to the reflexive blink and may even be more “strongly sensitive” to emotional state compared to the startle blink.

I find this argument, as it is currently presented, fairly weak. While there is overlap in these circuits, the spontaneous blink is far more removed from circuitry related to anxiety regulation than the autonomic response to startle stimuli. It would be helpful if the authors detailed the specific relationship between anxiety and the spontaneous blink circuit to demonstrate why they would expect a correlation and why it might be more strongly sensitive to emotional state as they do.

**Authors** - we agree with the reviewer that we had no reasons to expect that blink measure would be more sensitive and strong in the relationship with anxiety than startle, so we have canceled this both in the introduction and discussion. Concerning reviewer's observation that our argument on common circuitry of blink reflex (startle) and spontaneous blinking is fairly weak we do not agree, so we have detailed and made more compelling our assumptions on how anxiety may affect the two measures (spontaneous blinking and startle), in almost one page of the discussion, and along three lines of evidence (see below and text highlighted pg. 13):

"The two measures, startle reflex and BA, are necessarily similar for three reasons. First, blink amplitude measured by the integrated EMG (representing the overall spectral energy of muscle contraction) of the *Orbicularis Oculi* muscle is expected to be correlated with another measure of the same effector, such as, the eyelid shift leading to a VEOG signal proportional to the speed of eye closure. This may be analogue to the measure of the heart rate by means of the electrocardiogram versus the photopletismogram: two methods measuring the same psychophysiological parameter (i.e., heart rate). Second, the two measures share the same neural network underlying blink generation (see the most recent review: [7]), which includes the brainstem, basal ganglia, the trigeminal nerve, but of course they are not equivalent because one is a very fast defense blink reflex elicited by strong external stimulation (e.g., a shot or a flash or lightning), the other is a slow spontaneous and erratic blink controlled by internal cerebral processes indirectly modulated by external not locked stimulation. Third, we expected that anxiety level would have been positively correlated with both blink amplitude and blink rate as one important hub modulating blink activity is the brainstem, more precisely the reticular formation also termed ARAS (Ascending Reticular Activating System) important for modulating arousal at cortical level through the diffused serotoninergic system. One of the main features of anxiety is the hyper-arousal leading to insomnia, tiredness, jittery. Fear eliciting stimuli as well as anxiety, activate the amygdala, which in turn, through its central nucleus, activates the reticular formation, and this leads to both a potentiated startle reflex [1, 5] and higher blink rate [31]. In the present study we did not seek to demonstrate that the two methods are more or less correlated, instead we aimed to show that, in line with our hypotheses based on the known neural circuitry of the two measures, we expected an interesting convergence between the two blink amplitude methods in their capacity to correlate with state anxiety during both resting state and emotional stimulation conditions."

**Reviewer # 1 Q3 -** The authors state, “However, to the best of our knowledge, no study examined the interpersonal differences in eye blinking characteristics and their association with anxiety.” They should be aware that a study was published in March titled “ Emotional Blink Patterns: A Possible Biomarker for Anxiety Detection in a HCI Framework” (<https://doi.org/10.1007/s42979-025-03810-y>). I encourage the authors to incorporate this study into their literature review and interpretation.

**Authors -** as already written above, we have quoted this paper several times in both the introduction and the discussion (ref. # 9).

**Reviewer # 1 Q4 -** I am confused about why the authors did not make directional predictions about the expected correlation between the characteristics of the blink. It seems that if the theory was there to link the spontaneous blink with measures of anxiety, the authors would have expectations about the specific ways the blink would be correlated to these measures.

**Authors -** we thank the reviewer as we forgot to specify explicitly in what direction we expected the correlation of blink measures with anxiety. This could be implicitly inferred from startle reflex amplitude that was increased in high anxiety individuals, so we expected that blink amplitude and blink rate (known to be modulated by arousal levels) were positively correlated with anxiety levels. We have added the terms "positively correlated" in both the intro and the discussion sections. We have also added a sentence in the intro (pg.4) clarifying our predictions:

"More in detail, we expected that blink amplitude, similarly to startle amplitude, would be positively correlated with anxiety (greater anxiety, greater blink amplitude). Furthermore, we also expected blink rate, which has been shown to be modulated by fear [31] and anxiety [9], to be positively correlated with anxiety."

**Reviewer # 1 Q5 -** I have some concerns that this study was not planned a priori, but rather the data were accessed 3 years after they were collected and used to assess a second question. In addition to ethical concerns (e.g., does this study fall under the language of informed consent participants agreed to?), it also means the study wasn’t developed with the measurement of spontaneous eye blink in mind. For example, when collecting the spontaneous blink, it is standard to ask participants to remove contact lenses because those can impact the characteristics of the spontaneous blink. Was this done in this sample? In addition, because factors that affect the spontaneous blink (e.g. dopamine level) are sensitive to environmental conditions such as room temperature, these are usually controlled for. This should, at the very least, be noted as a limitation of the study.

**Authors -** concerning the concept of "study a-priori planned" we have already answered this question, the study was a-priori planned and the new measures and analyses were a-priori planned three years after the data collection for completely different aims and hypotheses from the original setting and acquisition, therefore this was a new study, and the raw data should be considered a convenience sample from which it was possible to extract the new measures (see also above and highlighted parts pg. 7 of the Ms.). We have also clarified that our informed consent (the standard form is written by the Ethics Committee) does not restrict the use of the data for one specific aim and study, see below (pg. 7).

"Furthermore, participants signed the standard informed consent suggested by our Psychology Ethics Committee for a generic use of their anonymized data, use that is not restricted to the specific hypothesis of the original experiment."

Concerning the procedure, it is important also for a good EEG recording to avoid contact lenses so we have added this sentence (pg.7):

"As a standard procedure of our EEG lab, participants with contact lenses were invited, before the recording session, to bring and use their glasses. Environmental temperature and background noise that could affect blink measures are typically controlled and stable in our dedicated psychophysiological laboratories."

**Reviewer # 1 Q6 -** I am not clear on the purpose or value of recording the blink during the emotional video clips, since the researchers ended up averaging across different emotions (including neutral). I agree with their assessment that this likely provided a more stable reflection of the blink, given the longer timeline. However, we know that tasks change the blink parameters, and the authors do not address this at all. Ultimately, it’s not clear how this part of the study helps us to understand the relationship between the spontaneous blink and anxiety, aside from showing that we see the amplitude relationship in both resting and task states.

**Authors -** we think we had addressed this issue in our discussion. Now, in the revision, we have further extended in several points the discussion on this issue. We better explained the rationale, we have also quoted the suggested paper on anxiety during emotional video-stimulation (ref. #9), see below (and pg. 15):

"In the present study we aimed to contrast two important conditions used in the literature typically in different experiments and contexts. Having in one session both resting state and visual stimulation allowed us to infer how the environment in two important ecological conditions influence blinking. Indeed one condition is passive characterized by mind wandering and self-reflection and stimulus rejection, the other condition is the opposite, being characterized by stimulus intake and blinking tracked by specific stimuli grabbing attention. In general, we found a stronger association between blinking characteristics and anxiety during the film clip watching than during the resting state condition. This is consistent with past study [9] (on blink rate only) reporting an interaction between anxiety and emotion during the presentation of emotional videoclips ".

Furthermore, we remind that in the original version of our manuscript we also tried to explain the difference observed in the two conditions by suggesting two possible mechanisms, (see bottom of pg.15):

"First, although each film clip was shorter, about 2 min, compared to the resting state, condition the 18 films overall resulted in 36 min of blink recording, thus providing a more stable and reliable blink measure with respect to the 5-min resting state recording. A second mechanism would be the reduction in unexplained interpersonal variance due to the focus on the visual channel and the repetition of similar visual stimuli. In the rest condition, without a focus on the external world, a broad range of erratic thoughts and emotions may occur by increasing variance between participants that is unrelated to the previously reported state of anxiety. Indeed, visual stimuli could narrow down the range of thoughts and, by limiting mind wandering, allowed a better representation of interpersonal differences in spontaneous blinking."

**Reviewer # 1 Q7 -** Similar to the introduction, I find the interpretation of why the spontaneous blink would be related to measures of anxiety to be fairly weak. The authors state: “We expected that the new measures derived from spontaneous blinking would have shown similar or even stronger effects as compared with startle amplitude.” and I’m not clear why it would be a stronger effect. Again, it makes sense that the amplitude of a startle blink would correlate to state anxiety, but not why the amplitude of the spontaneous blink would. This is especially true since the authors note that the mechanism is not equivalent (despite them talking about the similarity of the overlapping circuits).

**Authors -** we have already addressed this issue in our answer to Q1 and Q2, we have canceled the adverb "strongly" in both the introduction and discussion. Again see our response to Q2 and read Ms. pg.13 for a detailed explanation of the neural circuitry and common hub explaining how anxiety can modulate both spontaneous blink and blink-startle reflex.

**Reviewer # 1 Q8 -** In their conclusion, the authors argue that this could be an easy, non-invasive proxy of anxiety in participants, such as in “individuals at distance such as in telematic video transmissions.” However, they also note that “While BR and BRV can be easily recorded also by means of eye-trackers and video-recording, BA and BAV can be more easily measured by means of the VEOG trace from EEG recordings." If it’s only the blink amplitude that is informative about anxiety and blink amplitude needs some type of EEG/EOG measure, then that doesn’t seem that it can be used in the manner they are suggesting.

**Authors -** First of all**,** during the film session also BR and BRV were correlated with state anxiety thus under this specific condition, in addition to amplitude, also frequency analysis (BR and BRV) showed a significant correlation with anxiety. Second, after the revision, now in the Conclusions section we suggest that blink amplitude could be in principle measured by means of high speed video-recording: although this has not been demonstrated so far, there is room for future promising implementation of this new approach (see pg. 16):

"However, BA (and related BAV) could be in principle extracted from video-recording performed at high speed and high resolution by measuring the speed of eye closure, an index probably highly correlated with VEOG: this new method deserves to be further investigated in future studies."

**Reviewer # 1 Q9-** I encourage an in-depth review of this manuscript by someone whose native language is English. There are many grammatical errors and typos (e.g. the use of “addictive” rather than what I assume is “additive”, inconsistent use of past tense, use of “less” rather than “fewer”, etc.)

**Authors -** we thank the reviewer for the advice, we have amended the typos and made it thoroughly checked by a native English speaker.

**Reviewer # 1 Q10 -** I suggest a reorganization of the flow of the introduction. For example, it would make sense to present the basic definitions of the spontaneous blink and the startle blink before the literature on how these might relate to anxiety.

**Authors -** early in the introduction, pg. 3, now we have briefly described what is startle reflex so that the reader should be able to distinguish spontaneous blink from blink-startle reflex. See pg. 3 highlighted part:

"However, in comparison with the rare and recent use of Blink Rate (BR) in psychology, the most investigated index so far for studying emotions, anxiety and psychopathology has been the blink reflex, also termed startle reflex, a quick automatic defensive reflex represented by a very fast blink induced by a strong perceptual stimulation like a thunder, a shot or a flashlight."

---------------------------------------------------------------------------------------------------------------------

**Reviewer #2 Q1:**  Since eye movements can be captured with ease and non-invasively in naturalistic conditions, blink-metrics certainly offer real world application potential. BA and BR and their variations can also act as potential candidates for biomarkers;

**Authors -** we thanks the reviewer for the acknowledgement of the application potential of blink parameters as potential psychophysiological markers of anxiety.

**Reviewer #2 Q2:**  The experiment seems to have considered a sample having high and low ED in the absence of any healthy control. The correlation of BA with state-anxiety will be similar for healthy control also. It will also be similar for rest state with less value. However, during emotional film-clip viewing due to high or low arousal the BA variation will be more. But, again, this is true for healthy control also. Hence, can a threshold be established to distinguish between healthy control and anxiety affected with BA variation. Yes, it is true for State anxiety, the BA variation certainly counts. But the correlation found out is so less (<0.5) and the sample consists of only female candidates. BA will also vary from emotion to emotion individually. In emotional film viewing, how far it is convincing to take the average of BA variation. Rather, BA variation should be studied separately for +ve and -ve emotions.
But when it comes to Trait Anxiety, which is more stable and less sensitive to immediate stimuli, claiming a strong association from a modest correlation (-0.35) in a small, homogeneous sample feels premature.

**Authors -** we acknowledge that the sample is limited to female students, but our students were healthy as we excluded all participants with psychopathological or psychiatric issues. It is true that these healthy students were selected for high vs low levels of emotional dysregulation but they were within normal healthy levels of dysregulation parameters. Furthermore, sample size (overall n=50) is reasonable for a correlation design and is comparable to past research and statistical power which used blink measures. Indeed, we quoted Das et al., 2025, n= 44, and Maffei & Angrilli, 2019, n = 38). In the revised text we added (pg. 6 highlighted in yellow):

"...50 female healthy participants were selected out of a 422 students pool from the University of Padua. This sample is in line with sample size used in past research [9, 31] and with statistical power computed for blinking measures [31]."

"The original sample of fifty participants was recruited from a larger sample of healthy female students, indeed participants with high pathological levels of depression or anxiety were excluded [14]."

Concerning the correlation, our experience tells us that when you correlate two very different domains, the psychological (subjective measure of anxiety by the STAI) and the psychophysiological (blink measures) a correlation of 0.5 should be considered quite large. We agree on the limited value of the correlation found with Trait-Anxiety, a result that we have not further discussed. But not for the correlation with State-Anxiety: since the correlation index is also a measure of Effect size, according to statistical acknowledged reference (Kim, 2015 see ref.) a correlation of 0.5 or above is considered large effect size and r around 0.3 is considered medium effect size, both are not considered small. See commented on this at pg. 12 bottom:

"From another view, we can also consider the effect sizes of the links among our variables: indeed Person's correlation, in addition to the statistical significance which depends on sample size, is also an effect size, with r= 0.1-0.2 representing a small effect, r = 0.3 a medium effect, and r = 0.5 a large effect [26]. Thus, most of our effects ranged from medium (r= 0.288) to medium-large (r= 0.352) to large (r= 0.5 or higher).This provides further insight on the strength of our effects."

**Reviewer #2 Q3:**  BR and BRV can be more significant candidates as biomarkers for exploration of trait anxiety and for distinguishing between healthy control and anxiety affected. Again, these should be studied separately for +ve and -ve emotions.

**Authors -** studying blink measures in individuals with high trait anxiety vs normal healthy would be an interesting and natural prosecution of current research as in our study participants were not selected for trait anxiety levels. Concerning effects of positive and negative emotions on blink measures this has been investigated in our past quoted paper (Maffei & Angrilli, 2019) but only in normal healthy students. It would be interesting to extend research on emotional responses in high vs low anxiety individuals, this has been done in the new paper we quoted and suggested by Rev. n. 1, Das et al., 2025, but as written in a footnote, results of that study can not be compared with ours for methodological reasons (see footnote pg. 15):

"A more compelling comparison of the present results with the quoted study [9] is not possible for several methodological differences, especially because we measured the absolute blink rate along a continuum of anxiety levels, while the other study normalized blink rate within-subjects and analyzed emotion-related patterns in high vs low anxiety groups."

**Reviewer #2 Q4:**  Equation1) and 2) should be clearly explained along with significance of the coefficients.

**Authors -** thanks for the suggestion, we have clarified all the section with equations**,** Equations 1 and 2 and definitions of all terms, see pg. 10 all highlighted parts:

**"Equation 1.** A mixed model predicting participant j’s blink measurement (BM) when watching film i. $\beta_{0}$: overall intercept, $u_{0j}$: random intercept for each participant, $\beta_{1}$: fixed effect of STAI and $\varepsilon_{ij}$: residual error (unexplained variance) ${BM}_{ij}=\beta_{0}+u_{0j}+\beta_{1}{STAI}_{j}+\beta_{2}{Type}_{ij}+\beta_{3}{({Type}_{ij}\cdot STAI}_{j})+\varepsilon_{ij}$

**Equation 2.** A mixed model predicting participant j’s blink measurement (BM) when watching film *i*. $\beta_{0}$: overall intercept, $u_{0j}$: random intercept for each participant, $\beta_{1}$: fixed effect of STAI, $\beta_{2}$: fixed effect of film type effect, $\beta_{3}$: interaction effect between Type and STAI and $\varepsilon_{ij}$:residual error (unexplained variance)."

**Reviewer #2 Q5.** can it be justified in quantitative terms: “trait anxiety is mediated by state anxiety” with BA variation and BA as parameters? Summary of my observations are as follows:

• The resting state correlations tell us more about the inter-relatedness of blink metrics than anxiety per se.
• The findings during emotional stimulation seem more valid for State Anxiety, but even there, the statistical strengths aren't that sound.
• For Trait Anxiety, the associations are speculative at best without a larger, diverse sample and a more rigorous analytical design.
• The study has novelty and the level of enthusiasm is commendable, but some methodological gaps like lack of a control group and demographic diversity, modest sample size, and speculative generalizations limit the strength of its claims

**Authors -** we agree with the reviewer that the correlation of BA with trait anxiety is less consistent than the many correlations found with state anxiety, although the effect size (r= 0.354) can be considered medium and not small. However, we did not discuss much about this effect rather we focused on State Anxiety. As already written above to Reviewer #1, we had several significant correlations both during resting state and video clip presentation, and the effect sizes ranged from medium to large, thus we think our effects are not weak at statistical level. Indeed, the sample size were comparable (even larger) with those used in past research on blink (44 and 38 participants in the two quoted studies on blink rate, one of which also reported the expected sample size from an a-priori statistical power computation). We have also added a final sentence suggesting the need to enlarge the sample of participants with different demographic and clinical features, but here we need to highlight that most of basic research in psychology starts from the enrollment of the student community with the aim to enlarge samples with more specific features in subsequent investigations. Finally, we remind to the reviewer that all our participants were healthy and not pathological thus the control group was the sample of participants and we analyzed the correlation among all variables within this group rather than splitting the group as this would have required a different statistical approach (ANOVA and not Pearson's correlation).
