# Supplementary material for: The eye, a spy hole on human mind: Spontaneous blink rate and amplitude, and their variability, as new psychobiological markers of anxiety
Source: PLoS One. 2025 Dec 10;20(12):e0338262. doi: 10.1371/journal.pone.0338262 (PMC12694841; doi:10.1371/journal.pone.0338262)
Supplement: Attachment — Submitted filename: Response to Reviewers.docx [file pone.0338262.s004.docx]

Reviewer #2: The eye, a spy hole on human mind: spontaneous blink rate and amplitude, and their variability, as new psychobiological markers of anxiety: The title of the paper suits well with it's subject matter and appeals to students and researchers focused on noninvasive approaches to study mental health. Several eye parameters have been useful in the study of human emotions. Studies to identify biomarkers of affective states carries significance in the area of mental health research. However, limited available resources significantly hinder mental health research efforts. I appreciate the efforts of the authors to clarify the questions raised and justify their claims.

One suggestion as follows may be included in the manuscript: "explanation on the inclusion of the random intercept when overall intercept (beta0) is already present" for clarity.

The paper can be accepted.

Authors: we thank the reviewer for acknowledging our efforts to improve clarity of our Ms. As suggested we have added a sentence at pg. 10 (highlighted in bold) to clarify the "... inclusion of the random intercept...".

“For this purpose, mixed linear models predicting blink measurements (BR, BA, BRV and BAV) were conducted to account for the relationship between the 18 observations of each individual using subject as a clustering variable (random intercept) **addressing the statistical dependency among repeated observations within each subject**, and the STAI score as a predictor (see Equation 1).”
